# Dilution-Based Estimation of Intrinsic pH: A Proof-of-Concept in Plant Apoplastic Samples

**DOI:** 10.1021/acsomega.5c08356

**Published:** 2025-11-18

**Authors:** László Horváth, Tünde Alapi, Tímea Süli-Zakar, Zoltán Bozóki, Anna Szabó

**Affiliations:** † Department of Optics and Quantum Electronics, University of Szeged and HUN-REN-SZTE Research Group for Photoacoustic Monitoring of Environmental Processes, Szeged H-6720, Hungary; ‡ Department of Molecular and Analytical Chemistry, University of Szeged, Szeged H-6720, Hungary; § Institute of Plant Sciences and Environmental Protection, University of Szeged, Hódmezővásárhely H-6800, Hungary

## Abstract

Accurate determination of apoplastic pH is essential for understanding foliar gas exchange and nutrient transport in plants. However, direct pH measurement in apoplastic fluid is often limited by small sample volumes and technical constraints. We present a simple, dilution-based method to estimate the native pH of apoplastic fluid using the linear relationship between pH and the logarithm of the dilution factor. By extrapolating this relationship, the undiluted pH can be inferred without the need for titration or chemical additives. This approach preserves the sample’s native chemical equilibrium and enables robust estimation of pH in microliter-scale extracts. The method also provides an empirical buffer capacity indicator and may offer a practical alternative for apoplastic pH assessment in plant physiological studies.

## Graphical abstract

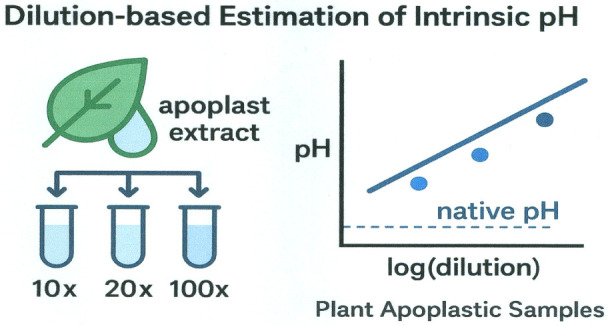

## Introduction

The plant apoplast acts as a mediating medium for gas exchange between the atmosphere and the foliage. Its acidity can determine the exchange characteristics of certain compounds. For example, in the bidirectional exchange mechanism of ammonia, apoplastic pH plays a key role. In the case of foliage, the compensation point potential used in exchange models is governed by the ratio of ammonium to hydrogen ion concentration.^1^ Therefore, knowledge of the hydrogen ion concentration, or pH of the apoplast is an essential input parameter in such models.

Yu et al.^2^ listed several methods for determining apoplastic pH, one of which is direct measurement in apoplastic fluid. However, a major limitation of this method is the small sample volume available for pH measurement. The extraction of apoplastic fluid is a complex procedure and typically yields only a small amount of sample, often insufficient for direct pH determination. The sample must therefore be diluted, but due to the buffer capacity of the apoplast, hydrogen ion concentration does not decrease in direct proportion with dilution. Estimating the original (undiluted) pH requires knowledge of the sample’s buffering capacity. This can be determined by various methods e.g., by CO_2_ titration,^3^ though their application generally involves complex procedures.

While microelectrodes allow pH measurement in very small volumes, their application to apoplastic fluid analysis is constrained. They are fragile, expensive, and prone to signal instability and drift, particularly in low-volume, weakly buffered biological samples. Calibration is also more complex and less reproducible than with standard electrodes. In contrast, conventional pH measurement is technically simpler, more robust, and better suited to routine analysis

In this brief communication, we present a new approach for estimating the native pH of small-volume apoplastic samples, using an extrapolation method based on the relationship between pH and dilution.

## Methods

### Sampling

Leaf sampling was carried out between 31 March and 9 May 2025 from a winter wheat crop. On each occasion, approximately three sets of 5 g leaf material were collected and stored frozen until processing.

### Recovery of Apoplastic Fluid

Several methods exist for extracting apoplastic fluid, with the most established for leaves being infiltration–centrifugation. For apoplastic fluid recovery, we applied the sample preparation method described by Husted and Schjoerring,^4^ with the modification that, due to the use of frozen samples, it was not feasible to cut small leaf segments (25 mm × 50 mm). Instead, significantly larger leaf pieces were used for extraction. As a result, no infiltration solution was required, and the samples contained no added solvents.

The recovery procedure was based on the technique described by Mattsson and Schjoerring,^5^ combined with the centrifugation method of O’Leary et al.^6^ No isotonic or buffer solution was used. Centrifugation was performed for 5 min at 1000 rpm and 5 °C. Visual inspection of the leaves postcentrifugation confirmed that most of the infiltrated liquid had been removed.

The relatively small sample size reflects the labor-intensive nature of the extraction procedure and the limited amount of apoplastic fluid obtainable from leaf tissue.

### pH Measurement

Before analysis, the samples were filtered using a syringe filter (FilterBio, Nantong, China; PVDF-L, 0.22 μm). The pH was measured using an InoLab pH 730p device with a Sentix 61 glass electrode.

### Calculation

A linear correlation and regression analysis was performed using the measured pH values and the dilution factors.

## Results

Depending on the available volume, the extracted apoplastic fluid was diluted 10-, 20-, 50- or 100-fold. The regression function used was pH = a × log_10_(D), where the slope a = dpH/d log­(D) can be regarded as an *empirical buffer capacity*, which is inversely proportional to the true buffer capacity. The latter is formally defined as β = dn/dpH, where *n* is the amount of equivalent strong base (or acid) added per liter of buffer solution. The regression lines are shown in Figure 1, while the results of pH measurements are illustrated in Table 1. For few samples, data were available at all dilution levels. Even with only three degrees of freedom, we found statistically significant correlations at the *p* = 0.1–5% level. The estimated pH of the undiluted samples (labeled as 1×) was derived from the y-intercept (b) of the fitted line, representing the extrapolated value at the undiluted state. The reciprocal of the slope is proportional to the buffer capacity. The values of b ranged from 0.22 to 0.50, with a median of 0.28 ± 0.073.

**Figure 1:**
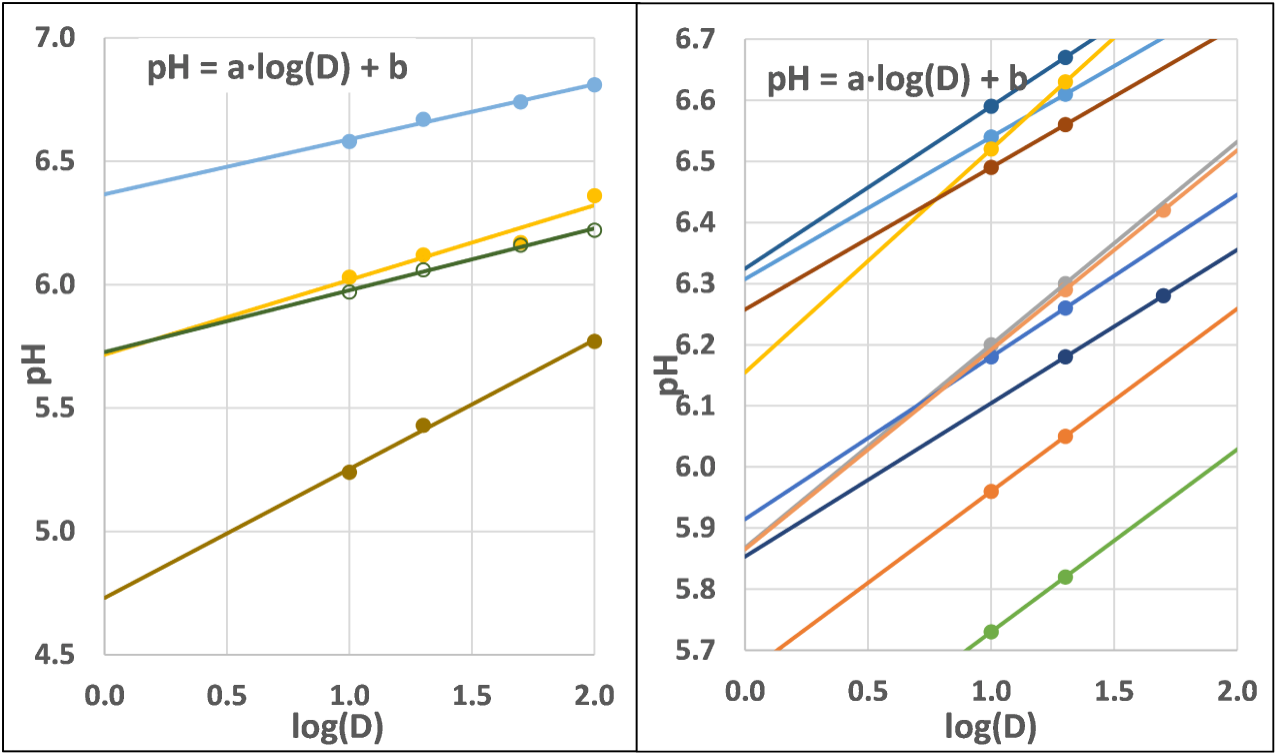
Variation in apoplastic pH as a function of dilution.

**Table 1:** Measured pH in Samples after Dilution (10×, 20×, 50×, 100×)^a^

date	pH	pH	pH	pH	pH	a	R^**2**^
	1×	10×	20×	50×	100×		
31.03.2025	6.31	6.54	6.61			0.233	
02.04.2025	5.66	5.96	6.05			0.299	
05.04.2025	5.87	6.20	6.30			0.332	
08.04.2025	6.15	6.52	6.63			0.365	
11.04.2025	5.91	6.18	6.26			0.266	
14.04.2025	5.43	5.73	5.82			0.299	
17.04.2025	6.32	6.59	6.67			0.266	
20.04.2025	6.26	6.49	6.56			0.233	
23.04.2025	5.72	6.03	6.12	6.17	6.36	0.302	0.908
26.04.2025	4.73	5.24	5.43		5.77	0.522	0.980
03.05.2025	5.85	-	6.18	6.28		0.251	
07.05.2025	5.73	5.97	6.06	6.16	6.22	0.250	0.994
08.05.2025	6.37	6.58	6.67	6.74	6.81	0.223	0.990
09.05.2025	5.87	-	6.29	6.42		0.327	
**mean**	**5.87**					**0.30**	
**median**	**5.87**					**0.28**	
**SD**	**0.43**					**0.077**	

a pH 1× indicates the extrapolated native pH of the samples prior to dilution.

As shown in Figure 1, in several cases the regression was based on only two data points, which is statistically questionable. Therefore, we assessed how the intercept (i.e., the estimated original pH) obtained from the multipoint regression compares with the value calculated using only the 10× and 20× dilutions from the same samples. The results are presented in Table 2, which illustrate the robustness of the proposed method. The magnitude of the difference is roughly comparable to the random error of the pH measurements.

**Table 2:** Comparison of the Estimated pH from Multi- and Two-Point (10×, 20×) Dilution from the Same Samples

multi point	10×, 20×	difference
6.37	6.28	0.09
5.72	5.73	–0.01
5.73	5.67	0.06
4.73	4.61	0.12

## Discussion

This dilution-based method enables pH measurement in apoplastic extracts even where only very small sample volumes (10–100 μL) are available. It requires no chemical reagents thus preserving the original chemical equilibrium. The principle of the measurement is simple, requiring only accurate pH determination No complex reagents or titration steps are needed. Based on the pH versus dilution curve, the native pH can be estimated even when direct measurement is not feasible.

A further limitation of our approach is that in some cases the regression was based on only two dilution points. We fully acknowledge that such regressions are statistically weaker compared to multipoint fits. This constraint arose primarily from the very limited sample volumes, which restricted the number of feasible dilution steps. Nevertheless, the comparison presented in Table 2 shows that the extrapolated pH values derived from two-point regressions were highly consistent with those obtained from multipoint regressions, with differences within the typical measurement error of the pH electrode. This agreement demonstrates that, despite their reduced statistical power, two-point regressions still provide reliable estimates of the undiluted apoplastic pH in cases where larger dilution series cannot be performed.

Another limitation of this study is the relatively small sample size, which inevitably reduces the statistical strength of the results. This constraint arose from the labor-intensive extraction process and the restricted availability of apoplastic fluid. Consequently, the present findings should be regarded as an initial proof-of-concept rather than a definitive statistical analysis.

A further limitation of the present study is the lack of independent validation, such as replication in a separate data set or by comparison with an alternative model. The absence of such validation lowers the overall confidence in the conclusions. While replication was beyond the scope of this proof-of-concept work, it should be prioritized in future studies.

A limitation of the method is that it is not standardized and not commonly used in the literature, making comparisons with other studies more difficult. Another potential concern is the effect of decreasing ionic strength during dilution on dissociation equilibria, as the apparent p*K*_a_ may shift.

A major limitation of the present study is the lack of direct comparison with established methods such as microelectrodes or CO_2_ titration. Such parallel measurements would certainly provide valuable validation of the method’s accuracy and a clearer demonstration of its advantages. However, due to the very limited sample volumes, it was not feasible to perform these comparisons in the current work. We therefore emphasize that testing the dilution-based approach against established techniques should be considered a priority for future studies. At the same time, the novelty of our method lies in its simplicity and feasibility in cases where conventional approaches are impractical, which makes it a potentially valuable complement to existing techniques.

However, the dilution inherently accounts for systematic changes in the physicochemical properties of the solution, including ionic strength and the associated shifts in apparent p*K*_a_ values. While ionic strength can significantly affect activity coefficients—and thereby the effective p*K*_a_—this influence manifests gradually and proportionally with dilution. As a result, the p*K*_a_ variation contributes consistently to the overall trend in measured pH values across the dilution series. Provided that the relationship between pH and log­(D) remains approximately linear (as observed in our data), the regression captures the cumulative effect of such changes. Consequently, the extrapolated pH value for the undiluted sample is not biased by the p*K*_a_ shift, since this shift is implicitly incorporated into the fitted function. This makes the approach robust even in cases where the ionic strength varies considerably across the dilution range.

## Conclusion

The method described provides a simple approach that leverages the relationship between pH and dilution. It appears applicable across the relatively broad pH range characteristic of the apoplast.

This study should be regarded as a proof-of-concept restricted to winter wheat. While the method shows promise, its broader applicability cannot be assumed at this stage. Testing across different plant species and under diverse environmental conditions will be essential to establish the general validity of the approach.

It will also be essential to validate this approach using independent data sets and across additional plant species before its general applicability can be established.

Furthermore, the empirical buffer capacity (i.e., the reciprocal of the slope *b*) could be compared with values obtained from established methods for buffer capacity determination, such as CO_2_ titration (Oja et al., 1999). Linearity could be verified by including undiluted samples at the beginning of the dilution series in future experiments.
